# A robust molecular probe for Ångstrom-scale analytics in liquids

**DOI:** 10.1038/ncomms12403

**Published:** 2016-08-12

**Authors:** Peter Nirmalraj, Damien Thompson, Christos Dimitrakopoulos, Bernd Gotsmann, Dumitru Dumcenco, Andras Kis, Heike Riel

**Affiliations:** 1 IBM Research—Zürich, Säumerstrasse 4, CH- 8803 Rüschlikon, Switzerland; 2 Department of Physics and Energy, University of Limerick, Limerick V94 T9PX, Ireland; 3 Materials and Surface Science Institute, University of Limerick, Limerick V94 T9PX, Ireland; 4 Department of Chemical Engineering, University of Massachusetts, Amherst, Massachusetts 01003-3110, USA; 5 Electrical Engineering Institute, École Polytechnique Fédérale de Lausanne (EPFL), CH-1015 Lausanne, Switzerland; 6 Institute of Materials Science and Engineering, École Polytechnique Fédérale de Lausanne (EPFL), CH-1015 Lausanne, Switzerland

## Abstract

Traditionally, nanomaterial profiling using a single-molecule-terminated scanning probe is performed at the vacuum–solid interface often at a few Kelvin, but is not a notion immediately associated with liquid–solid interface at room temperature. Here, using a scanning tunnelling probe functionalized with a single C_60_ molecule stabilized in a high-density liquid, we resolve low-dimensional surface defects, atomic interfaces and capture Ångstrom-level bond-length variations in single-layer graphene and MoS_2_. Atom-by-atom controllable imaging contrast is demonstrated at room temperature and the electronic structure of the C_60_–metal probe complex within the encompassing liquid molecules is clarified using density functional theory. Our findings demonstrates that operating a robust single-molecular probe is not restricted to ultra-high vacuum and cryogenic settings. Hence the scope of high-precision analytics can be extended towards resolving sub-molecular features of organic elements and gauging ambient compatibility of emerging layered materials with atomic-scale sensitivity under experimentally less stringent conditions.

## Abstract (web summary)

Single-molecule-terminated scanning probes typically operate under ultra-high vacuum conditions at low temperatures. Here, the authors show that tips functionalized with C_60_ can image single-layer graphene and MoS_2_ with high definition in a liquid environment at room temperature

## Graphical abstract

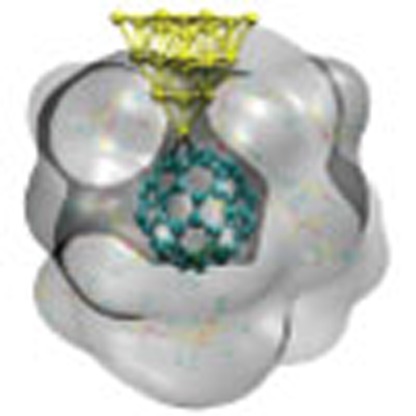

Sensing objects below the resolution limit of the eye began with the advent of water-based microscopes in 2000 BCE. Following a series of ground-breaking advances, instrument development eventually led to the modern electron and scanning probe microscopes which have not only advanced resolution capability but have enabled new research disciplines from quantum cryptology to single-molecular nanotechnology. Recently, high-resolution transmission electron microscopy^1^, scanning tunnelling microscopy (STM)^2^,^3^,^4^ and scanning tunnelling spectroscopy^5^ have made possible the analysis of a new class of ultra-thin materials. The atomic-scale structure, extrinsic doping, bonding states and chemical composition can be precisely measured for single-atom-thick electronic materials from the two-dimensional (2D) form of carbon, graphene^6^ to more recent transition metal dichalcogenides (TMDs)^7^ with potential for triggering a new wave of 2D nanodevice technologies.

However, preserving the surface integrity of single-atom-thick materials outside of ultra-high vacuum (UHV) settings, and extracting information with highest possible resolution, where each atom is directly exposed to contaminants is not trivial. Yet the payoff is immense. To take one illustrative example, it would allow rapid fingerprinting of a vast body of theoretically predicted 2D layered materials^8^ and simultaneously verify the ambient compatibility of such novel materials^9^ and 2D patterned structures^10^. A known alternative to vacuum to protect air-sensitive surfaces, is the liquid–solid interface in which a STM can be operated. This field of research has an almost 30-year history, from early reports on achieving atomic resolution on solid surfaces immersed in water^11^, liquid nitrogen^12^ and acidic solution^13^ environments, observation of molecular dynamics^14^, decoding molecular layer-underlying surface epitaxial relationship^15^, capturing oxidation catalysis reactions^16^ and in investigating through high-resolution STM images the supramolecular chemistry of molecules^17^ and pattern formation during molecular self-assembly^18^ at the liquid–solid electrical interface with a non-functionalized metal probe. More recently it has been demonstrated that by terminating the apex of a scanning probe (including STM and AFM) with a single molecule, it is possible to further the limits of spatial resolution and enhance chemical contrast of the low-dimensional materials under study^19^,^20^,^21^,^22^,^23^,^24^,^25^,^26^. However, these single-molecule-terminated scanning probes have been mainly demonstrated to operate at UHV in a temperature scale ranging from 4 to 100 K (refs 20, 21, 22, 25, 27, 28). The protocols for engineering such molecular probes, in particular bonding effects between the molecule and the metal-apex, have been documented in detail^22^,^24^,^27^,^28^,^29^,^30^. Conversely, the benefits of the molecular probe have not been fully exploited under standard laboratory conditions, mostly because of instabilities in molecule–metal coupling at room temperature. In the present work, we demonstrate that by engineering and operating a single-molecule-terminated Au STM probe in high-density liquids, it is possible to control random fluctuations of the molecule at the metal apex, maintain a clean interface protected from ambient contaminants thereby resulting in a robust single-molecular probe with prolonged lifetime.

## Results

### Electron tunnelling in a high-density liquid environment

**Figure 1:**
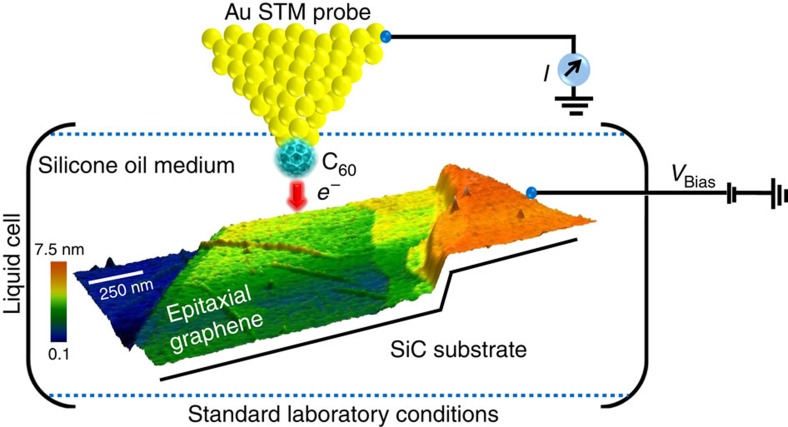
Description of the liquid-based STM experimental design. Schematic of the liquid-cell setup with the single-molecule-terminated Au tip (connected to an external current pre-amplifier circuit) positioned over an epitaxial graphene sample (on which the bias voltage is applied). The entire liquid cell holds an electrochemically inert and high-density silicone oil at room-temperature conditions. The liquid cell is based on Teflon.

The central concept of our experiment is depicted in Fig. 1, a metallic (Au) STM probe functionalized with a C_60_ molecule is brought close to the surface (epitaxial graphene on SiC^31^) held in a Teflon-based liquid cell filled with silicone oil ([-Si(CH_3_)_2_O)-]_*n*_). C_60_ geometry permits easier lateral manipulation and transfer to the tip from the surface at ambient conditions than, for example, CO, and the protocol for C_60_ transfer is well established^19^,^22^,^24^,^32^. Single C_60_-terminated STM tips are routinely used to probe organic materials and C_60_ clusters anchored on metal STM tips have also been demonstrated previously, to investigate low-dimensional surface defects^33^. Such molecular cluster-based tips are relatively robust (loss of a single molecule at the apex can be compensated by its nearest neighbour) but interpretation of the observed chemical contrast improvements is not trivial. The mechanism of electron tunnelling from the C_60_-terminated Au probe through a non-polar liquid medium into the conductive graphene layer may be modelled as tunnelling through a vacuum gap where the tunnelling current (*I*_tunnel_) is exponentially dependent on the distance (*Z*) between the probe and the conductive surface using an effective energy barrier height (φ_eff_) as shown below,


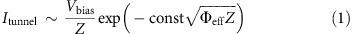


However, the effective energy barrier height (φ_eff_) has been shown to fluctuate for STM measurements at the conductive liquid–solid electrical interface^34^. In addition to fluctuations related to φ_eff_, the tunnelling probability (both elastic and inelastic) during STM measurements in polar liquids can be influenced by the barrier dielectric response. To exclude these effects, it is necessary to work in a liquid that is electrochemically inert, has a low affinity towards the sample and a low vapour pressure. The dense molecular structure of silicone oil prevents moisture build-up on the solid surface, protects air-sensitive samples^12^,^35^ and minimizes tip and surface contamination^36^. Note that during the tunnelling process (irrespective of bias polarity), the liquid can be squeezed out of the gap and this holds for both polar^37^ and non-polar tunnel barriers^12^,^35^,^38^.

### Engineering and testing the robustness of the molecular probe

**Figure 2:**
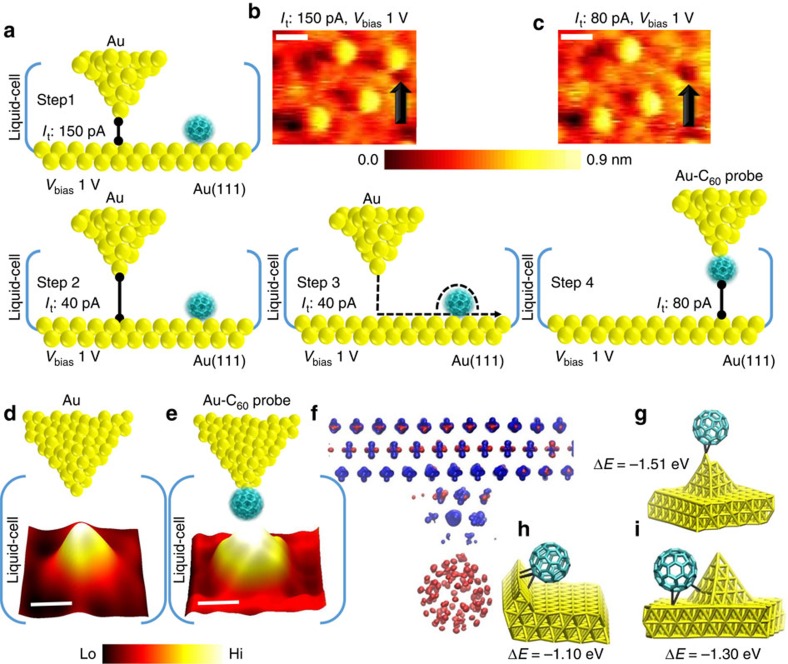
Fullerene terminated STM probe formation, analysis and electronic structure. (**a**) Schematics detailing the steps involved in formation of a molecular probe by controlled transfer of a surface confined molecule to the apex of the Au probe. (**b**) Constant-current STM image of isolated C_60_ molecules adsorbed on Au(111). The molecule marked by the black arrow is selected for the transfer. (**c**) Imaging the same area after the lateral manipulation transfer experiment. The scale bars in **b** and **c** are 1.1 nm. (**d**,**e**) Control experiments in which the individual molecule on the surface is imaged using a Au probe (**d**) and C_60_-terminated Au probe (**e**), showing different structural lineshape with the C_60_ probe revealing submolecular structure. (Tunnelling parameters for data shown in **d** and **e** are *I*_tunnel_ (*I*_t_)=50 pA, *V*_bias_ (*V*_b_)=0.5 V. The scale bars in **d** and **e** are 0.8 nm. (**f**) Computed electron density surface for the molecular probe in silicone oil showing electron density maps of the occupied states at the top of the valence band (coloured blue, calculated over the energy range of −1 V up to the Fermi energy, set to 0 V) and unoccupied states at the bottom of the conduction band (coloured red, calculated in the energy range of 0 V to +1 V). The isovalue used is 0.05 and the atoms are removed for clarity. (**g**–**i**) Electronic structure calculations of representative bonding sites of the C_60_ molecules on nanostructured gold. Au–Au and Au–C distances below 3.2 and 3.0 Å are drawn as sticks and C–C distances below 2.0 Å are drawn as sticks. ΔE is the computed C_60_–Au bond energy.

Single C_60_-terminated metal STM tips have been fabricated in the past through lateral manipulation and transfer of a surface bound C_60_ molecule to a metal apex under cryogenic conditions^22^ and later applied to resolve intramolecular details of molecules^19^ and applied in atom-scale engineering of single-molecule contacts^24^. We attempt a challenging lateral manipulation technique to engineer a C_60_ functionalized Au probe in a liquid environment at room temperature. For this we first locate an isolated C_60_ molecule on Au(111) surface in silicone oil liquid cell and the specific molecule was later transferred to the metal tip by controlled lateral manipulation as shown from the schematic steps (step 1–step 4) in Fig. 2a. The tunnel current set point is reduced by nearly 30% during transfer attempt in comparison to the set point applied during normal imaging experiments. To verify whether the lateral manipulation approach was successful, the same region is scanned again. The target molecule indicated by the black arrow in the STM image (Fig. 2b) and after the lateral manipulation (Fig. 2c) experiment in the presence of the liquid medium, serves as a first proof of the transfer of a C_60_ molecule from the surface to the Au tip. However, we did not observe any immediate apparent change in the structure of the near-lying C_60_ molecule when imaged with a C_60_–Au probe as shown in Fig. 2c, which was recorded at a high speed (scan rate of 30.5 Hz, tip velocity of 15.3 μm s^−1^ and 256 × 256 lines per scan area). This is in contrast to previous low-temperature STM studies of C_60_-terminated tungsten tips^22^, where directly after termination of the surface bound molecule to the metal apex resulted in visualization of the molecular orbitals of the C_60_ when imaged over Au adatoms, which served as sharp reverse imaging sites. In our study, upon reducing the scan speed (scan rate of 1.51 Hz, tip velocity of 0.7 μm s^−1^ and 512 × 512 lines per scan area), we obtained an improved visualization of the structural change incurred on the surface bound C_60_ molecule (Fig. 2e), when compared with the featureless structural shape imaged previously for the same molecule with a non-functionalized Au tip (Fig. 2d). After passing this control test, the molecular probe (C_60_–Au STM tip) was then directly transferred to a liquid-cell holder with the epitaxial graphene sample encompassed in silicone oil. Here again we conduct an additional control test to confirm the presence of the molecular probe, which is a reverse imaging experiment at local defect sites on graphene. The observed structural changes were comparable to early C_60_-terminated STM-based reverse imaging studies on defects on a graphite surface^39^, which further validates the presence of the C_60_ molecule at the Au tip–apex. (see ‘Methods' section and Supplementary Fig. 1, Fig. 2a,b). Note: In total, we have conducted 135 attempts to fabricate the C_60_ molecular STM probe through lateral manipulation, of which ∼80 were successful. This nearly 65% success rate includes tests where we were in the process of finding the optimal tip–sample height distances and bias energy ranges. Generally the failed attempts were due to incidents where the bias energy (≥1.8 V) and/or the current set point (≥250 pA) was too high, during the manipulation attempt, suggesting that the molecule was displaced rather than transferred. Note: The optimal bias energy range and tunnel current set point values apply only during manipulation experiments, the molecular probe after fabrication can be operated in a bias energy range of −2 V to +2 V and under a tunnel current set point range of a few pA to ∼1.5 nA. However, the best imaging on 2D materials was obtained at low bias and low tunnel current set points and this also extended the lifetime of the molecular probes.

Similar transfer experiments in the absence of the silicone oil liquid medium had a much lower success rate of 10% and even the few molecular probes that we were able to synthesize did not last for more than a few scans under dry conditions, indicating the importance of the encompassing liquid medium in reducing the fluctuations of the molecule at the Au tip–apex. To further quantify the bonding mechanism of the C_60_ molecule to the Au tip, we rely on quantum mechanical models (representative structures shown in Fig. 2g–i, also see Supplementary Methods 1 for details of the density functional theory (DFT) calculations) which indicate that the C_60_ molecule exhibits a significant preference of (0.35±0.03) eV per molecule for bonding to low-coordination defect sites rather than terrace sites on Au and reveal that the strongest covalent character for C_60_ attachment occurs at the apex site (by contrast, terrace sites form predominantly van der Waals attachments to C_60_).

### Atom-scale metrology of graphene systems

**Figure 3:**
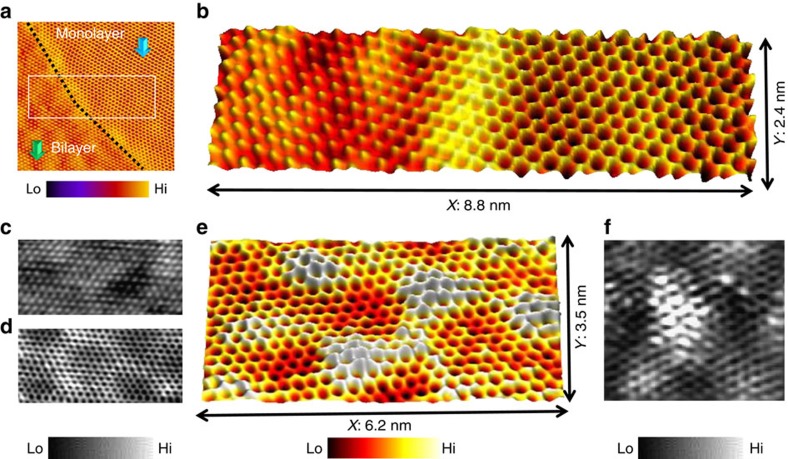
Atomistic metrology of epitaxial graphene in liquid environment. (**a**) Wide-angle constant-current STM image revealing monolayer and bilayer graphene regions. The black line indicates the interface between the regions (Tunnelling parameters: *I*_t_=85 pA, *V*_b_=1.1 V, scan size: 9.4 nm × 8.7 nm). (**b**) High-resolution constant current STM image showing the bilayer and monolayer regions within a single frame. (Tunnelling parameters: *I*_t_=65 pA, *V*_b_)= 1.3 V). (**c**,**d**) Constant-current STM image showing atomically clean lattice of bilayer and monolayer graphene, respectively. (Tunnelling parameters for **c**: *I*_t_=100 pA, *V*_b_=0.9 V, scan size: 5.2 nm × 2.5 nm; and for **d**: *I*_t_=100 pA, *V*_b_=1.1 V, scan size: 5.8 nm × 2.7 nm). (**e**) STM image highlighting local protrusions and depressions in single-layer graphene (*I*_t_=100 pA, *V*_b_=0.9 V, scan size: 6.2 × 3.5 nm). (**f**) High-resolution constant-current STM image of point defect on monolayer graphene lattice (*I*_t_=25 pA, *V*_b_=0.3 V, scan size: 2.7 nm × 2.5 nm). Scan speed parameters for **a**, **c**, **d** and **f** were maintained at 8 Hz at 512 × 512 lines per scan and for **b** and **e** were recorded with a scan speed of 2 Hz at 512 × 512 lines per scan.

Figure 3a is a large-area STM image acquired on a graphitic region. The interface (marked by the black dotted line) separates the monolayer region (marked by the blue arrow) and bilayer region (marked by the green arrow). The area marked exactly at the interface (indicated by the white rectangle in Fig. 3a) is projected in a three-dimensional format as shown in Fig. 3b. The content of the imaged structure (Fig. 3a,b) is in agreement with previous UHV-STM experiments performed on similar graphene systems at 4 K (ref. 40) and high-resolution AFM studies at room temperature^41^. Zooming into the separate regions indicates clearly the bilayer structure (Fig. 3c) and monolayer graphene with a honeycomb-like architecture (Fig. 3d). A three-dimensional representation of a similar monoatomic graphene lattice (Fig. 3e) highlights the atomic hillock-and-valley structures, signifying that these 2D materials are not perfectly planar and can have differences in the local corrugations. Imaging on a separate region on the monoatomic graphene lattice, we also observed point defects exhibiting a two-fold symmetry (Fig. 3f) as previously observed on monolayer graphene imaged in a UHV-based STM using non-functionalized metal tips^42^. The occurrence of corrugations and point defects, appearing as protrusions on the atomic lattice, is dependent not only on the synthesis mechanism but relates strongly with the registry of these materials to the underlying surface and its roughness^43^. The coupling of the C_60_ molecule to the metal–apex within the silicone oil medium is key to the stability of these molecular probes under ambient conditions. Control experiments where the molecular probes were used to image graphene (with similar bias and tunnel current set points) but under dry-conditions lasted for only a few scans (five to eight frame scans at a rate of ∼15 s per frame, after which the molecule was lost, confirmed through experiments conducted on isolated C_60_ molecules and reverse imaging^33^ over lattice defects on graphene, see section 1 Supplementary Information). This is in contrast to the longer lifetime of the molecular probes in silicone oil (∼60 frame scans of comparable scan areas at a rate of ∼15 s per frame, and in a few cases longer than 60 frame scans when applied for imaging over graphene regions free of defects and protrusions).

### Quantifying bond lengths and molecular fluctuations

**Figure 4:**
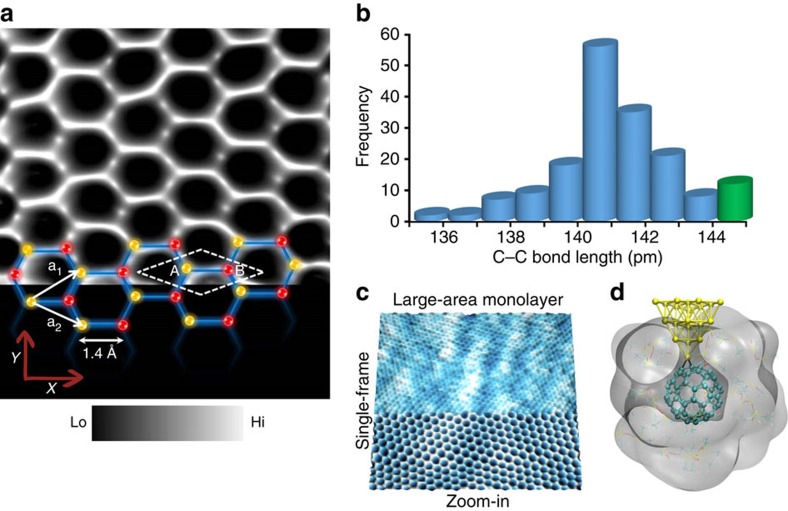
Liquid-stabilized molecular probe for bond length quantification. (**a**) High magnification constant-current STM image within a graphene monolayer region acquired using a single C_60_ molecular probe showing the local arrangement of the atomic lattice of graphene (*I*_t_=200 pA, *V*_b_=0.5 V, scan size: 1.7 nm × 1.2 nm). The yellow and red spheres point to the two equivalent carbon atoms present within a primitive unit, indicated by the white dashed rhombus and lattice vectors a_1_ and a_2_, within a monoatomic *sp*^2^ bonded network of graphene. (**b**) Statistical distribution of C–C bond lengths measured using a C_60_-molecular probe on a monolayer of graphene spanning an area of ∼200 nm^2^. The statistical distribution shown in **b** is based on systematic analysis over a large-area monolayer (90 nm nm × 125 nm) followed by zoom-in (11.3 nm × 10.3 nm, on specific locations to extract the C–C bond distance values (**c**). The scanning parameter remain constant, (*I*_t_)=155 pA, (*V*_b_)=0.6 V. The upper C–C bond length values displayed in the histogram coded in green were measured using the molecular probe on atomic hexagons near the vicinity of point defects on monolayer graphene. Scan speed parameter for **a** and **c** was 2 Hz and 12 Hz at 512 × 512 lines per scan, respectively. (**d**) Snapshot of the final simulated tip structure obtained after 50 ns of equilibrated room temperature molecular dynamics of the C_60_-functionalized gold tip in silicone oil. Silicone oil molecules within 5 Å of C_60_ are shown as lines with oxygen atoms coloured red, hydrogen white, carbon cyan and silicon yellow, and space-filling surface is overlaid (radius scale 1.2, density isovalue 1.0 and grid spacing 0.5—generated using visual molecular dynamics (VMD) software). More distant silicone oil molecules are omitted for clarity.

Figure 4a is a STM close-up view of the hexagonal honeycomb architecture. The disparity in the bond lengths connecting the monolayer of carbon atoms can be directly visualized and quantified, thus highlighting the capability of the molecular probe to operate in liquids. We calculated the mean bond length connecting two adjacent carbon atoms within an atomic hexagon to be 1.41 Å, consistent with previous high resolution STM reports performed under cryogenic conditions^44^. The measured bond length is double the atomic radius of carbon (0.7 Å). Factors such as substrate curvature, topological defects and surface corrugations can induce strain, leading to variations in the experimentally measured bond lengths. The upper limit of the bond length values we report (coded in green in the histogram, Fig. 4b) was mainly measured near point defects on the graphene lattice, particularly in the vicinity of a topological defect region (similar to the constant current STM data on a point defect in Fig. 3f). This is consistent with previous theoretical calculations predicting an asymmetric bond length distribution in the graphene lattice at room temperature^45^ and with previous high-resolution transmission electron microscopy analysis of C–C bond length fluctuations in single-layer graphene^46^.

To better understand the role of silicone oil in the formation of a stable molecular probe, we performed classical molecular dynamics simulations to examine the motion of C_60_ on the metal–apex in the presence and absence of the liquid. Single C_60_ dynamics on the tip–apex is expressed as root-mean-square fluctuations in the C_60_ centre of mass, averaged over 4,000 structures sampled (every 10 ps) during the final 40 ns of 50 ns of equilibrated room-temperature dynamics. The molecular dynamics model cell was based on DFT calculations of C_60_ bonding to an Au apex site via two Au–C bonds of length 2.2 Å (Fig. 4d). The C–Au bond force constants of 55 N m^−1^ are estimated from DFT by shifting the C_60_ molecule 0.25 Å closer and nearer to Au in steps of 0.05 Å, consistent with previous reports^30^. Figure 4d is a snapshot of the final computed structure of the C_60_-functionalized Au probe in silicone oil. The computed C_60_ dynamics on the Au probe revealed significant reduction of C_60_ motion on the Au tip–apex in silicone oil. There is a three-fold damping when silicone oil covers the molecular probe (see Supplementary Methods 2 for more details of the molecular dynamics simulations).

### Tuning atom-by-atom imaging contrast

**Figure 5:**
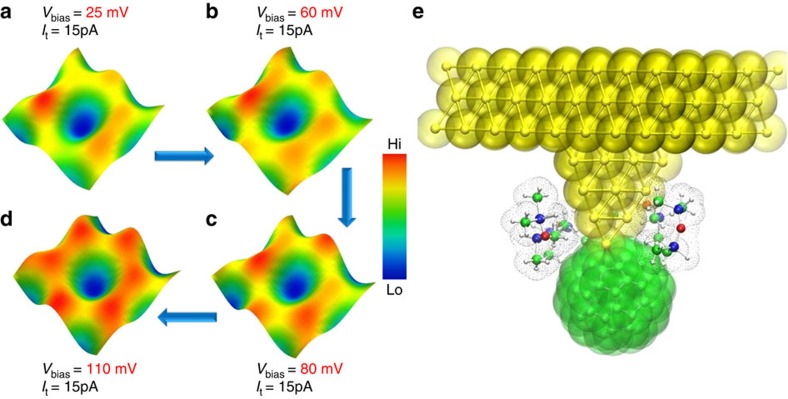
Tuning atom-by-atom imaging contrast with a single-molecular probe. (**a**–**d**) Series of three-dimensionally represented constant-current STM images over an atomic hexagon recorded under incremental bias steps at a fixed tunnel current set point. The positions of all six carbon atoms within a hexagonal ring are directly resolved as shown in **d**. Scan speed parameters for **a**–**d** are maintained at 2 Hz at 512 × 512 lines per scan. (**e**) The calculated electronic structure of the molecular probe complex with silicone oil molecules coordinating the C_60_-gold interface. Atoms are shown in ball and stick representation with space-filling spheres overlaid (dotted surface used for solvent). Au atoms are coloured gold, C atoms are green, Si atoms are blue, O atoms are red and H atoms are coloured white. The computed solvent structure is shown in an alternative orientation in Supplementary Fig. 7, together with more details of the local and extended solvent structure around the probe.

Having established a route to resolve with high spatial clarity the covalent bonding sequence at a fixed bias energy, we then posed the question, is it possible to control the imaging contrast at a single-atom level? Figure 5a–d is a series of constant current STM images acquired over a single atomic hexagon spanning an area of 4.2 Å^2^ represented in a three-dimensional format, imaged at a fixed tunnel current set point (15 pA) and increasing the bias energy in steps. We observe a marked improvement in the ability of the molecular probe to identify the exact location of the atoms (Fig. 5a–d) within a six-membered carbon hexagonal ring. The location of the atomic sites can be controllably revealed and masked by increasing and decreasing the bias energy at fixed tunnel current. This level of control and ability to tune the imaging contrast, permits direct visualization of the electronic variations in embedded atoms, in this case the inter-atomic bonds within a single hexagon. We believe the high spatial information content upon terminating the Au tip with a C_60_ molecule and bias-dependent control of contrast can be attributed to the corresponding alterations in the tip states, magnitude of the bias energy and the resulting changes in the spatial orbital overlap between the molecular probe and monoatomic graphene. The local electrostatic interactions^47^ between the molecule and the graphene surface is also a factor that contributes to the enhancement in imaging contrast. To gain a deeper quantitative understanding of the impact of tunnelling parameters on the STM images in a liquid medium, it is requisite to establish a first principle-guided study of electron tunnelling between a molecular probe and graphene lattice in the presence of a silicone oil mesh similar to previous studies on electronic tunnelling through polar liquids^48^.

Although we have been able to previously image the atomic lattice of single-layer graphene in liquids using non functionalized metallic tips^36^, with resolution comparable to the data shown in Fig. 3d, it has not been possible to achieve the resolution and imaging control shown in Fig. 4a and Fig. 5a–d using a bare metallic Au tip in liquids at room temperature. Such high resolution imaging where the exact atomic positions can be identified allows better quantification of bond atomic distances in 2D crystals that is closer to the expected theoretical estimates. To check for any electronic interference from the silicone oil medium in the observed variations in imaging contrast, we performed dispersion-corrected electronic structure calculations on the C_60_-terminated Au probe in the presence of silicone oil molecules. The C_60_–Au electronic structure does not change when the solvent layer is included (Fig. 5e).

### Probing atomic landscape of 2D MoS_2_

**Figure 6:**
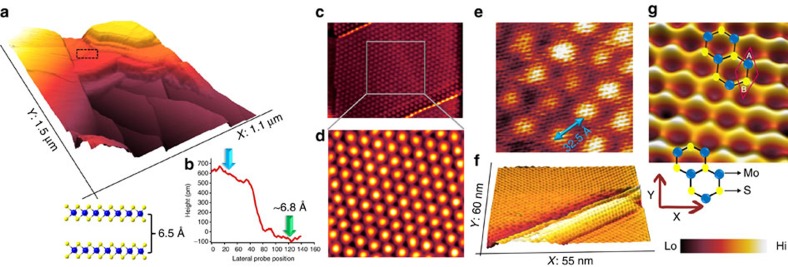
Imaging the atomic lattice of 2D MoS_2_ on Au(111) in liquid using a molecular STM probe. (**a**) Large-area STM image of MoS_2_ grown on Au(111), imaged using a C_60_-terminated Au probe in silicone liquid environment (*I*_t_=650 pA, *V*_b_=1.1 V). A line section analysis at the MoS_2_–Au interface shows a height difference of ∼6.8 Å (**b**) indicating a region of single layer MoS_2_. The probe is then positioned on the region marked by the black rectangle in **a** and a higher-resolution constant current image is obtained (**c**) which shows the atomic structure of MoS_2_ and the bare Au surface at the edges. (*I*_t_=450 pA, *V*_b_= 0.8 V, scan size: 90 nm × 70 nm). (**d**) Zoom-in of the region indicted by the grey square in **c**, revealing the moiré superstructure (*I*_t_=450 pA, *V*_b_=−0.8 V, scan size: 32 nm × 33 nm). (**e**) Atomic structure of the moiré region (*I*_t_=125 pA, *V*_b_=−0.25 V, scan size: 7.5 nm × 7.3 nm) with a period of 32.5 Å. (**f**) STM image of a line-defect type atomic structure observed in the MoS_2_ domain (*I*_t_=180 pA, *V*_b_=+0.5 V). (**g**) Atom-resolved STM image of the hexagonal lattice structure of single-layer MoS_2_ with a measured lattice constant of 3.2±0.1 Å (*I*_t_=40 pA, *V*_b_=+0.3 V, scan size: 2.7 nm × 2.5 nm). The scan speed parameter for **a**, **c** and **e** was 6 Hz and for **d**, **f** and **g** it was 2 Hz at 512 × 512 lines per scan.

In addition to studying the lattice structure of monoatomic graphene with a molecular STM probe in liquids, we demonstrate that this robust probe can also be extended to provide a quantitative analysis at a single-atom level of the structural composition of other 2D layered materials such as TMDs. For this, we use a MoS_2_ sample grown by chemical vapour deposition (CVD) on Au(111) (for further details on growth protocols, see ‘Methods' section). The 2D-MoS_2_ is an electronic material gaining immense interest as a transistor channel material^49^, in biomolecular sequencing^50^, information storage^51^ and in analytical chemistry^52^ for its built-in semiconducting nature and atomic structure. The as-grown MoS_2_–Au(111) sample is then transferred to the liquid-cell holder filled with silicone liquid and imaged using a single C_60_–Au STM probe engineered using protocols identical to those used to image graphene systems. Figure 6a is a large-area STM image showing regions of MoS_2_ layers grown on Au(111) terraces. Single-layer regions are identified by measuring a height of ∼6.8 Å at the MoS_2_ layer and gold interface (within the region marked by the black dotted rectangle in Fig. 6a), as shown in the line section analysis (Fig. 6b). This is consistent with the expected layer thickness of TMD materials that ranges from 6.5 Å to 7.1 Å with a layer of metal atoms trapped between two layers of chalcogen atoms (atomic model, Fig. 6b). The measured layer thickness is also in agreement with previous low temperature STM reports on CVD grown MoS_2_ (ref. 53) imaged using a non-functionalized metal probe. Positioning the molecular STM probe in an interface region between single-layer MoS_2_ and the gold surface reveals the moiré superstructure that stems from the lattice mismatch between the underlying Au(111) surface and the as-grown MoS_2_. A close-up view of this moiré superstructure presented in Fig. 6d and the atomic structure of the moiré patterns is further resolved by zooming within the same region as shown in Fig. 6e. The periodicity of the moiré patterns is measured to be 32.5 Å (Fig. 6e) and the presence of line-type surface defects was also observed (Fig. 6f) consistent with recent STM studies of single-layer MoS_2_ epitaxially grown on Au(111) surface^54^. A spatially magnified constant-current STM image of the honeycomb type MoS_2_ atomic structure is shown in Fig. 6g. The position of the S atoms in the hexagonal structure is better resolved by the molecular STM probe than the Mo atom sites suggesting that the electron density is dominated by top-positioned S atoms^55^. On the basis of several such high-resolution images captured at low scan speeds (1–4 Hz at 512 × 512 lines), we were able to measure a MoS_2_ lattice constant of (3.2±0.1) Å, consistent with previous STM reports under UHV conditions using a non-functionalized STM probe. Coupled with the graphene data, this quantitative information on MoS_2_ indicates that a single C_60_-terminated STM tip can be reliably applied for atom-by-atom analytics of 2D nanomaterials, even when operated in liquids at room temperature.

## Discussion

Taken collectively, the scanning tunnelling microscopic observations and the molecular simulations provide compelling evidence for the damping of C_60_ motion at the probe metal–apex in silicone oil, resulting in a stable molecular probe. Given the growing interest in analysing atomic materials under practical conditions^9^,^10^,^41^,^56^, our findings provide a significant step forward in atom-mapping technology in liquids at room temperature, which can be applied to examine lattice geometry, ambient compatibility and probe the impact of solvents on next generation of 2D semiconducting solids^57^. However, a deeper understanding of the influence of rotational motion of the molecule at the probe–apex will be required before these findings can be fully extended to resolve the intramolecular architecture of organic molecules with an assortment of heterogeneous chemical bonds. To address these open issues, it will require bold efforts in the future in a liquid environment, to decode the orientation of the molecule at the probe–apex^19^ to establish a clearer interpretation on the origin of the visualized enhancement of information content from scanning probe images.

## Methods

### C_60_ solution synthesis

C_60_ molecules in powder form (purchased from Sigma-Aldrich, 99% purity) were solubilized (bath sonication 30 s) in toluene, and a solution with 0.1 mM concentration was prepared. One millilitre of this solution was drop-casted on a Au(111) surface. The excess solvent was blown dry using N_2_ gas and the sample was placed in a liquid cell and immediately covered with silicone oil. The silicone oil used in this study having a chemical formula of ([-Si(CH_3_)_2_O)-]_*n*_), where *n*=22, was purchased from Sigma-Aldrich. The CAS number is 63148-62-9.

### STM tip preparation

The tips (Au) used in this work was just mechanically cut with hand-held pliers, followed by rinsing in acetone (5 min bath sonication) and isopropyl alcohol (2 min bath sonication), blow drying with N_2_ gas (until dry) and followed by annealing at 50–55 degrees Celsius for 30 min in vacuum oven.

### Fabrication and analysis of the molecular STM probe

We compared the current–voltage curves recorded using non-functionalized Au tips and C_60_-terminated STM tips (see Supplementary Fig. 1) and thus confirm (based on the evolution from metal-metal contact to a molecular signature in the IV curve) that a C_60_ molecule is adsorbed on the Au STM probe apex. The imaging is then temporarily halted, the molecular STM probe is withdrawn and the Au(111) sample is replaced by the epitaxial graphene on SiC sample within the liquid cell and the molecular probe is brought back into contact with the surface. An additional step to check whether the molecule is still anchored to the apex in between scans of the graphene surface is through reverse imaging in liquids. Where the molecular probe is raster scanned over a sharp defect feature acting as a secondary site on the graphene surface. This is usually a topological defect that acts as a surface tip array which then reflects the image of the molecule adsorbed on the Au tip apex. One such STM image is shown in Supplementary Fig 2a and Fig. 2b, where the point defects on the graphene surface aid in imaging the molecule on the tip. The underlying graphene architecture is visible and the C_60_ molecular shape is reflected similar to Supplementary Fig. 2a.

In addition to the lateral manipulation experiment, we have also attempted vertical manipulation, through which the molecule on surface is transferred to the tip. For this, we place the Au tip on top of the C_60_ molecule, and vary the bias voltage when keeping the tip–sample height distance constant (fixed tunnel current set point). However, for the current work, we report only the molecules transferred using the lateral manipulation approach. The success of both these experiments, lateral and vertical manipulation, is strongly dependent on the low mobility of the underlying molecule and this is achieved by trapping the molecules in a high-density liquid such as silicone oil. To remove all other possible sources of environmental fluctuations, the measurements were conducted in next-generation noise-free laboratories, which offer vibration isolation together with precise temperature and humidity control. The specifications used include, active mechanical damping of the platform supporting the STM equipment down to 0.5 Hz, mechanical vibration with velocity less than 500 nm s^−1^ (*x*,*y*,*z*) below 16 Hz, electromagnetic fields with flux density less than 5 nT RMS in an integral spectrum between 0 and 625 Hz and temperature stability of 0.1 °C h^−1^ with fluctuations of 0.5 °C per day.

### Instrument and materials details

The STM measurements were conducted in constant-current mode using Nanoscope IIIa, E-scanner, Digital Instruments. A low-current pre-amplifier circuit capable of ∼1 pA sensitivity was used. The lateral STM tip drift rate is ≤1 nm min^−1^ in silicone oil medium. The STM instrument is allowed to scan with bare Au tips on lithographically designed calibration samples (Ted Pella Inc, 677-STM) for 6–8 h to reduce scanner hysteresis before mounting molecular STM probe for actual measurements on graphene. Nanoscale calibration of the scanner was performed using a freshly cleaved highly oriented pyrolytic graphite surface (*x*, *y* axes) and a Au(111) on mica substrate (*z* axis). A Teflon-based custom-built liquid-cell setup was used to support the sample substrates.

### Graphene synthesis

Graphene was synthesized by epitaxial growth by thermal decomposition of hydrogen etched 4H_SiC(0001) surface in a furnace.

### MoS_2_ growth on Au(111)

The monolayer molybdenum disulfide MoS_2_ samples have been obtained by CVD method on Au/mica substrates. The growth process is based on the gas-phase reaction between MoO_3_ and high-pure sulfur evaporated from solid phase. Crucible containing ∼5 mg MoO_3_ (≥99.998% Alfa Aesar) with Au/mica substrates was loaded into a three-zone furnace with a 32 mm outer diameter quartz tube. Located upstream from the growth substrates, a second crucible contained ∼350 mg of sulfur (≥99.99% purity, Sigma-Aldrich). Ultrahigh-purity argon (Ar) was used as the carrier gas. CVD growth was performed at atmospheric pressure. The recipe of MoS_2_ growth is as follows: ramp the temperature to 300 °C and set for 10 min at 200 s.c.c.m. of Ar flow, ramp to 700 °C with 50 °C min^−1^ rate and set for 10 min at 10 s.c.c.m. of Ar, cool down to 500 °C and further open the furnace for rapid cooling with the increasing of Ar flow to 200 s.c.c.m. As a result, monolayers MoS_2_ flakes of the order of 1 μm have been grown on Au/mica substrates.

### Data availability

The data that support the findings of this study are available from the corresponding author on request.

## Additional information

**How to cite this article:** Nirmalraj, P. *et al*. A robust molecular probe for Ångstrom-scale analytics in liquids *Nat. Commun.* 7:12403 doi: 10.1038/ncomms12403 (2016).

## Supplementary Material

Supplementary Information Supplementary Figures 1-7, Supplementary Table 1, Supplementary Methods and Supplementary References.

Supplementary Movie 1 Silicone oil damping of molecular motion. Movie shows the damping of translocation motion of C60 molecular motion as a result of encompassing liquid medium.

Supplementary Movie 2 Orientations of C60 near tip Apex. The movies show computed orientations of C60 obtained by sampling over 500 equally-spaced MD structures (every 20 ps) during the final 10 ns of dynamics. A green line is drawn between two arbitrary points through the centre of the C60 molecule and the tilt angles of that line trace the time history of C60 rotational orientations sampled during the calculations.

Supplementary Movie 3 Rotational states of C60 in vaccum. Movie shows three times more rotational states sampled by the probe in vacuum.
