# Supplementary material for: A robust molecular probe for Ångstrom-scale analytics in liquids
Source: Nat Commun. 2016 Aug 12;7:12403. doi: 10.1038/ncomms12403 (PMC4990633; doi:10.1038/ncomms12403)
Supplement: Supplementary Information — Supplementary Figures 1-7, Supplementary Table 1, Supplementary Methods and Supplementary References. [file ncomms12403-s1.pdf]

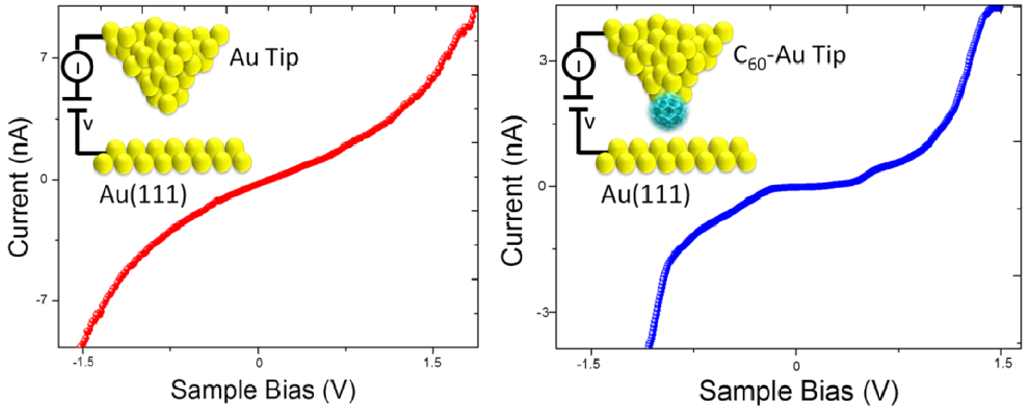

Supplementary Figure 1. Current-voltage curves. The bare Au probe on Au(111) surface (red curve) and corresponding curve recorded when a C_{60} molecule is in between the metal-metal junction (blue curve) in the case of the C_{60}-terminated STM probe.

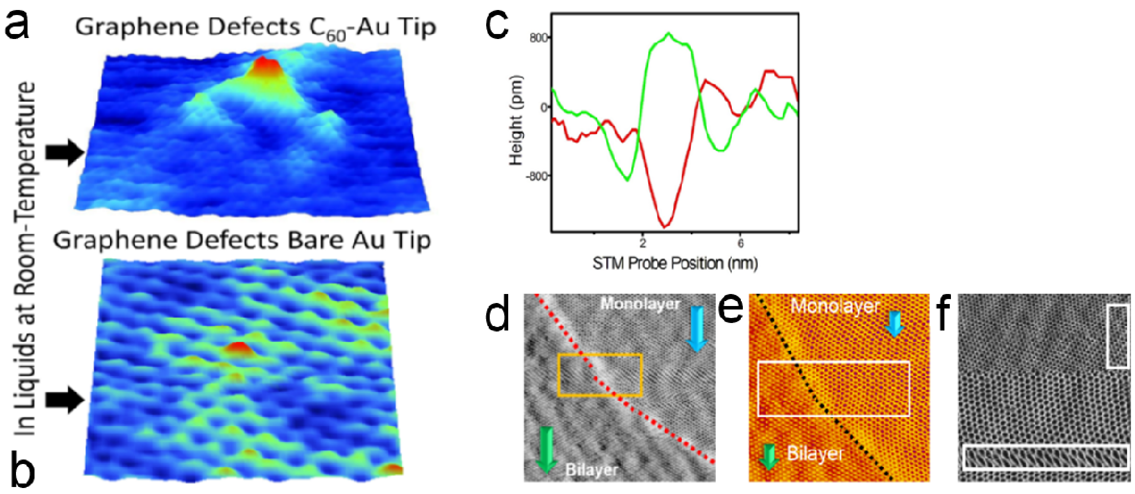

Supplementary Figure 2. Imaging protocols using a molecualar probe in liquids. (A-B) Imaging with a reverse imaging technique using graphene surface defects of C_{60} anchored on Au apex. I_t = 1.25 nA, V_b = 0.2 V. (C) STM scan profile when imaged over a target molecule (Fig 2b main manuscript) as shown in green line and profile of the same area (Fig 2C main manuscript) shown in red line after the target molecule is transferred to the tip. (D) STM image over a bilayer-monolayer graphene region where the scan speed is changed in the middle of the frame to 28 Hz and after a few seconds the scan rate is lowered to 4 Hz. This change in scan rate in between scans was used as a test to check for any improvements in the information content from the STM images. (E) STM image of a zoomed in area from panel D. STM image shown in section E was recorded with a fixed scan rate of 8Hz. (F) STM image recorded over a monolayer region before the piezo tube scanner was stabilized, which reveals imaging artifacts as outlined

in the white box in section F. All the high resolution images reported in the main manuscript and in the supplementary section were recorded after letting the scanner image for around 6-8 hours to minimize hysteresis and tip drifts.

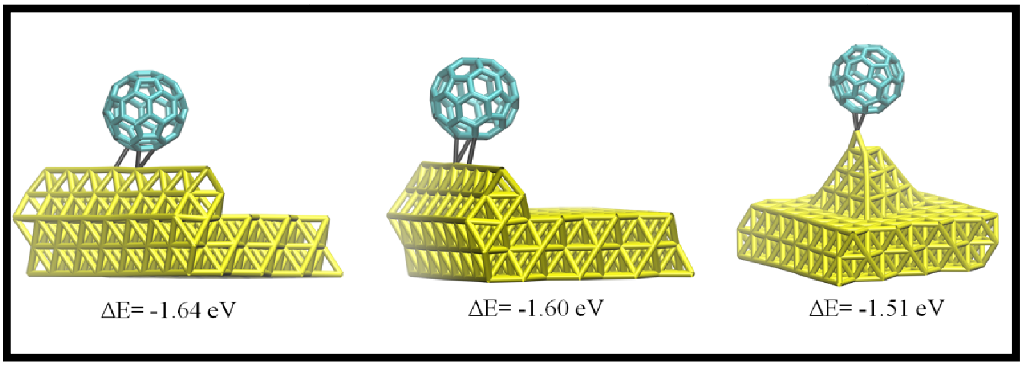

Supplementary Figure 3. C_{60} bonding to defect sites on Au tips. Au-C distances below 3.0 Å are drawn as black lines.

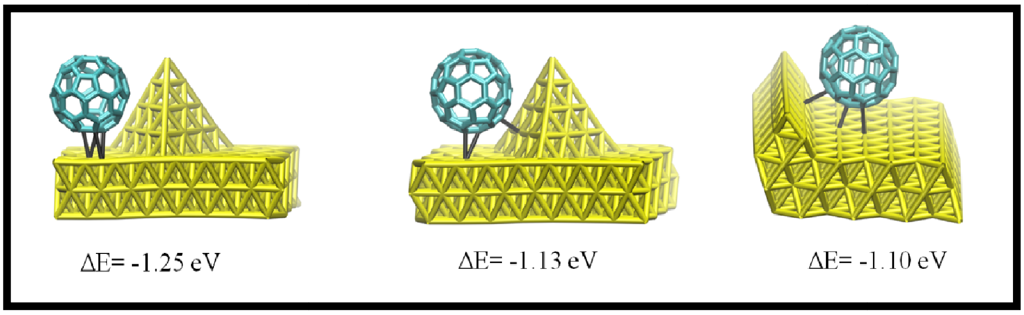

Supplementary Figure 4. C_{60} bonding to terrace sites on Au tips. Au-C distances below 3.0 Å are drawn as black lines.

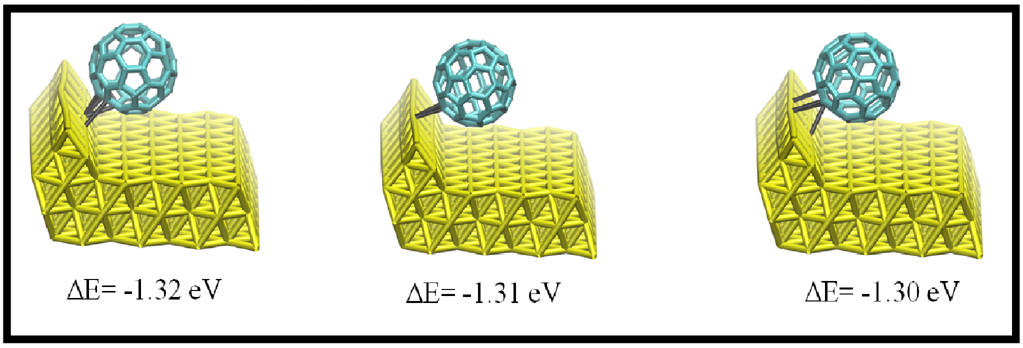

Supplementary Figure 5. Transitional bonding modes of C_{60} that are partially on defect and partially on terrace sites. Au-C distances below 3.0 Å are drawn as black lines.

The bonding of C_{60} to the gold tip is most covalent in character at the Au tip apex site, as determined using the Grimme D3 correction [1] to estimate the van der Waals contribution to C_{60}-

Au adsorption. We use the metric ρ to characterize the nature of the C_{60}-gold structures, where ρ is defined as the ratio of the covalent bond energy to the total binding energy, $\rho \equiv E_{\text{electronic}}/(E_{\text{electronic}} + E_{vdW})$ or alternatively $\rho \equiv \Delta E_c/(\Delta E_c + \Delta E_p)$. The covalent bond energy is estimated using the ΔE_c values above in Supplementary Table 1 and Supplementary Figure 3-Figure 5, ignoring van der Waals contributions, *i.e.*, the physisorption component, ΔE_p. A value of $\rho \approx 1$ indicates a covalent Au-C chemical bond while $\rho << 1$ indicates predominantly van der Waals binding and so physisorption of C_{60} [2]. On the terrace and mixed terrace/defect sites (Supplementary Figures 4 and 5) we calculated ρ values ranging from 0.35 to 0.39. Only the purely defect sites (Supplementary Figure 3) have $\rho > 0.5$, with ρ values ranging from 0.59 to 0.73. The highest value of $\rho = 0.73$ is found for C_{60} adsorbed at the Au apex (structure with ΔE_c = -1.51 eV in Supplementary Figure 3), indicating that the most covalent interaction occurs at the gold tip apex site.

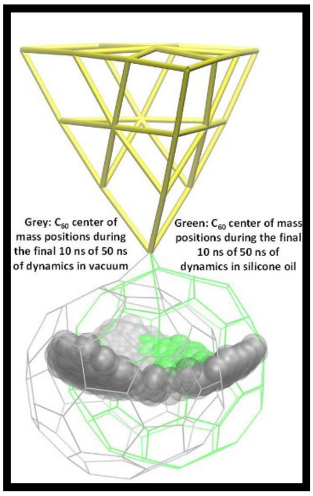

Supplementary Figure 6. Molecular dynamics simulations show that fluctuations in C_{60} position decrease three-fold in silicone oil. The green and black spheres mark centre of mass positions sampled in silicone oil and in vacuum. The Au tip is shown as gold sticks and representative snapshots of C_{60} in silicone oil and vacuum are drawn in green and black sticks. The computed C_{60} dynamics on the Au tip reveal significant damping of C_{60} motion when silicone oil is used as the encompassing medium. The motion of the C_{60} unit, as measured from root mean square fluctuations (RMSF) of the C_{60} centre of mass, drops from 2.7 ± 0.6 Å to 0.9 ± 0.2 Å when silicone oil covers the C_{60}-functionalised Au tip. Statistically identical RMSF values were obtained in additional control simulations with the ensemble switched from constant-pressure to constant-volume and simulations in which the number of 22-unit silicone oil polymer molecules in the encompassing medium was halved from 40 to 20. The damping effect of silicone oil on the C_{60}-functionalized Au tip is much larger than previously calculated for C_{60} physisorbed on the planar Au(111) structure. [3] In that case, C_{60} did not form a chemically-bound molecular probe on gold but instead just adsorbed through van der Waals forces, and the C_{60} RMSF values were respectively 3.5 ± 1.3 Å and 2.4 ± 0.6 Å in vacuum and oil.

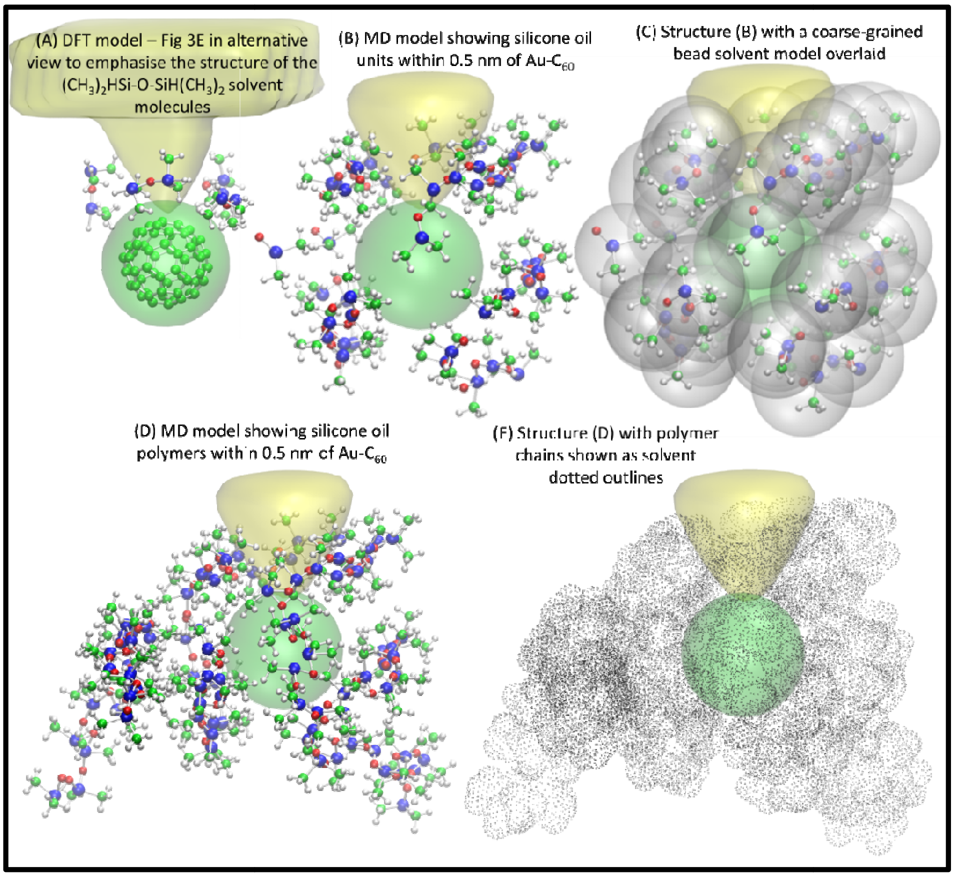

Supplementary Figure 7. Evolution of the model physics showing how the DFT model in panel (A) was generated from MD models of the extended silicone oil structure, with panels B-E showing a representative MD structure in a variety of representations to emphasise respectively (B) near-probe solvent units, (C) the tight packing of solvent units together and around the probe, (D) near-probe solvent chains, and (E) the shape of the near-probe solvent cloud. The gold tip and C_{60} molecule are shown as outline surfaces (gold and green respectively) and solvent molecules, where drawn explicitly, are shown in ball-and-stick representation. C atoms are coloured green, Si atoms are blue, O atoms are red and H atoms are coloured white. More distant solvent molecules are omitted for clarity.

The MD simulations show that the C_{60} molecule can rotate freely as it approaches the gold tip, as shown in movie Supplementary Movie 2. These rotations occur approximately three times faster in vacuum (see Supplementary Movie 3) than in the presence of silicone oil, which may explain why we see a significant damping of C_{60} center of mass root mean square fluctuation (1.8 Å → 0.7 Å) even prior to the formation of the chemical bonds between C_{60} and the most reactive tip apex site (at which point the presence of the two covalent bonds restricts rotation to one plane as the gold-bound hexagon remains parallel to the Au(111) surface). The silicone oil damps C_{60} motion by restricting both atomic positional fluctuations and full body rotations.

Supplementary Table 1. Chemisorption of C_{60} on Au tips

Binding site	Chemisorption energy, ΔE_c (eV)
Defect	-1.51 ± 0.04
Terrace	-1.16 ± 0.05

Chemisorption energies ΔE_c are calculated by comparing the electronic energies of the complex with the isolated surface and isolated molecule. Values shown are averages over the calculated binding geometries, six on defect sites (Supplementary Figure 3) and three on terrace sites (Supplementary Figure 4) calculated using two surface models. Three transitional structures were found corresponding to mixed defect/terrace binding models with calculated chemisorption energies of -1.31±0.01 eV (Supplementary Figure 5). The structure-averaged standard errors are <50 meV for each binding site, indicating a significant preference of 0.35 eV for adsorption at defect ridge/apex sites with a computed standard error of 0.03 eV in the set of 18 defect *vs.* terrace pair wise differences.

Supplementary Methods

Electronic Structure Calculations

Quantum mechanical models provide the structure for the C_{60}-Au molecular probe used in the classical molecular dynamics simulations described in section 4 below. These electronic structure calculations show three important features: (1) C_{60} molecules exhibit a mild preference of 0.35±0.03 eV/molecule for adsorption at low-coordination tip sites rather than terrace sites on Au (Fig. 1C-E in the main text and Table S1 and Supplementary Figures 3-5 b). (2) Dispersion-corrected electronic structure calculations indicate more significant covalent character for C_{60} attachment to low-coordination tip sites, in particular the lowest coordination apex sites, compared with terrace sites that form predominantly van der Waals attachments to C_{60} (see below). (3) Silicone oil molecules do not change the electronic structure of the C_{60}-Au complex (Fig. 3e in the main text). Taken together, these data strongly support the inference from the STM data (based on the tip stability and imaging precision obtained in the experiments) of single C_{60}-termination of nanostructured Au STM (single-atom apex) tips. Supplementary Table 1 shows computed chemisorption energies for C_{60} bonding to gold nanostructures (apex and ridge tip features, and terrace sites), and the corresponding computed structures are shown in Supplementary Figures 3-5. The density functional theory (DFT) electronic structure calculations were performed using the VASP program [4]. The periodic surface models were described using periodic plane wave DFT with the GGA-PBE functional [5], projector augmented wave (PAW) pseudopotentials [6] with a plane wave cut-off of 400 eV and a vacuum spacing of approximately 3 nm in the direction normal to the gold surface. The molecule-surface complexes were calculated using periodic boundary conditions. Molecule-surface binding energies were converged to below 100 meV using a 441 k-point grid. Chemisorption energies (ΔE_c) were calculated by comparing the electronic energies of the complex with the isolated metal and molecule.

Molecular dynamics simulations

Fully atomistic molecular dynamics simulations were performed using the NAMD code [7]. The CHARMM force field was used [8] with literature parameters for gold [9] and silicone oil [10], to model the stability of the molecular probe (C_{60}-functionalised Au tip) in silicone oil. The gold tip was modelled using a slab of Au(111) with a tetrahedral Au_{20} [11] surface feature on top. The

electronic structure of the molecular probe is described above in Supplementary Methods 1 above. The silicone oil solvent is described using 40 polymer chains each containing 22 [-$Si(CH_3)_2O$)-]$_n$ repeat units (n=22), similar to the experimental value of n~25. Simulations were performed at room temperature using a two femto second time step and structures were sampled for 50 nanoseconds of molecular dynamics, following minimisation and one nanosecond of thermalisation and equilibration. Gold atoms were constrained to their starting positions and kept neutral in the simulations. Single C_{60} dynamics on the tip-apex is expressed as root-mean-square fluctuations (RMSF) in the C_{60} centre of mass, averaged over 4000 structures sampled (every 10 ps) during the final 40 ns of 50 ns of equilibrated room-temperature dynamics. The error bars (±) are estimated by block averaging of ten, 4-ns blocks from the computed trajectories. Supplementary Figure 7 and Supplementary Movie 1 show how the silicone oil damps the motion of the C_{60} molecule, reducing three-fold its sampling of space below the tip (which is the region of space in which the C_{60} images the contact substrate, *e.g.*, graphene). The MD model cells were based on density functional theory (DFT) calculations (section S2) of C_{60} bonding to an Au apex site via two Au-C bonds of length 2.2 Å. C-Au bond force constants of 80 kcal/mole/$Å^2$ (55 N/m) were estimated from DFT by shifting the C_{60} molecule 0.25 Å closer and nearer to Au in steps of 0.05 Å, consistent with previous reports [12]. Control MD simulations using alternative C-Au bond force constants of 60, 90 and 100 kcal/mole/$Å^2$ gave identical results, within the error of the time-averaged C_{60} RMSF values.

Additional control simulations were performed of C_{60} dynamics on the Au tip, in the absence of the two specific pairwise C-Au bonds. Such models take into account that the tip is a dynamic nanostructure which may alter its shape during experimental timescales, and approximate early stage and transitional periods of dynamics in which C-Au bonds could be forming and reforming, perhaps due to Au adatom migration or damage caused during imaging. In these control simulations we use two alternative CHARMM-type potentials, one non-bonded [9] and the other a Morse-type non-pairwise bonded potential [13] and two alternative Au geometries (the Au_{20} cluster planted on a Au(111) slab as described above, and an isolated Au_{20} cluster). These models showed marginal damping of C_{60} motion in silicone oil, with the time-averaged RMSF values dropping from 1.8 ± 0.9 Å to 0.7 ± 0.2 Å for C_{60} rolling around on Au_{20} and from 1.2 ± 0.7 Å to 0.7 ± 0.1 Å for C_{60} adsorbed at the Au_{20}/Au(111) interface in a structure similar to that reported by Stadler *et al.*[14]. The two force fields [9,13] gave almost identical results, with the mean RMSF and errors differing by less than 0.1 Å. In these control simulations the mean RMSF decreases in silicone oil but the distributions overlap (0.9–2.7 Å in vacuum *vs.* 0.5–0.9 Å in oil and 0.5–1.9 Å in vacuum *vs.* 0.6–0.8 Å in oil). The large effect of silicone oil obtained for the covalently bound C_{60} on Au (2.1–3.3 Å in vacuum *vs.* 0.7–1.1 Å in oil) is more consistent with the experimentally measured improvements in STM imaging described in the main text, and is consistent with the C_{60} chemisorption energies on the Au tip in the range of -1.1 to -1.6 eV and significant covalent character of the C_{60}-Au apex structure obtained in the electronic structure calculations described in Supplementary Methods 1.

Supplementary References

1 Grimme, S., Antony, J., Ehrlich, S. & Krieg, H. A consistent and accurate ab initio parametrization of density functional dispersion correction (DFT-D) for the 94 elements H-Pu. *J Chem Phys* **132** (2010).

2 Geranton, G., Seiler, C., Bagrets, A., Venkataraman, L. & Evers, F. Transport properties of individual C-60-molecules. *J Chem Phys* **139**, (2013).

3 Nirmalraj, P. *et al.* Nanoelectrical analysis of single molecules and atomic-scale materials at the solid/liquid interface. *Nat Mater* **13**, 947-953 (2014).

4 Kresse, G. & Hafner, J. Abinitio Molecular-Dynamics for Liquid-Metals. *Phys Rev B* **47**, 558-561 (1993).

5 Perdew, J. P., Burke, K. & Ernzerhof, M. Generalized gradient approximation made simple (vol 77, pg 3865, 1996). *Phys Rev Lett* **78**, 1396-1396 (1997).

6 Blochl, P. E. Projector Augmented-Wave Method. *Phys Rev B* **50**, 17953-17979 (1994).

7 Phillips, J. C. *et al.* Scalable molecular dynamics with NAMD. *Journal of Computational Chemistry* **26**, 1781-1802, (2005).

8 MacKerell, A. D. *et al.* All-Atom Empirical Potential for Molecular Modeling and Dynamics Studies of Proteins†. *The Journal of Physical Chemistry B* **102**, 3586-3616, (1998).

9 Heinz, H., Vaia, R. A., Farmer, B. L. & Naik, R. R. Accurate Simulation of Surfaces and Interfaces of Face-Centered Cubic Metals Using 12−6 and 9−6 Lennard-Jones Potentials. *The Journal of Physical Chemistry C* **112**, 17281-17290, (2008).

10 Liu, Y. Z., Wu, M., Feng, X. Z., Shao, X. G. & Cai, W. S. Adsorption Behavior of Hydrophobin Proteins on Polydimethylsiloxane Substrates. *J Phys Chem B* **116**, 12227-12234, (2012).

11 Gruene, P. *et al.* Structures of neutral Au-7, Au-19, and Au-20 clusters in the gas phase. *Science* **321**, 674-676, (2008).

12 Nadine, H. *et al.* Force and conductance during contact formation to a C 60 molecule. *New Journal of Physics* **14**, 073032 (2012).

13 Wright, L. B., Rodger, P. M., Corni, S. & Walsh, T. R. GoIP-CHARMM: First-Principles Based Force Fields for the Interaction of Proteins with Au(111) and Au(100). *J Chem Theory Comput* **9**, 1616-1630, (2013).

14 Stadler, R., Kubatkin, S. & Bjornholm, T. An ab initio study of the field-induced position change of a C-60 molecule adsorbed on a gold tip. *Nanotechnology* **18**, doi:Artn 165501 (2007).
